# Non-catalytic roles for TET1 protein negatively regulating neuronal differentiation through srGAP3 in neuroblastoma cells

**DOI:** 10.1007/s13238-016-0267-4

**Published:** 2016-04-25

**Authors:** Jie Gao, Yue Ma, Hua-Lin Fu, Qian Luo, Zhen Wang, Yu-Huan Xiao, Hao Yang, Da-Xiang Cui, Wei-Lin Jin

**Affiliations:** School of Life Sciences and Biotechnology, Shanghai Jiao Tong University, Shanghai, 200240 China; Institute of Nano Biomedicine and Engineering, Department of Instrument Science and Engineering, School of Electronic Information and Electronic Engineering, Shanghai Jiao Tong University, Shanghai, 200240 China; National Center for Translational Medicine, Shanghai Jiao Tong University, Shanghai, 200240 China; Department of Experimental Surgery, Tangdu Hospital, Fourth Military Medical University, Xi’an, 710038 China; Clinical Stem Cell Research Center, Renji Hospital, School of Medicine, Shanghai Jiao Tong University, Shanghai, 200127 China

**Keywords:** methylcytosine dioxygenase, TET1, srGAP3, neuronal differentiation, neuroblastoma cells

## Abstract

The methylcytosine dioxygenases TET proteins (TET1, TET2, and TET3) play important regulatory roles in neural function. In this study, we investigated the role of TET proteins in neuronal differentiation using Neuro2a cells as a model. We observed that knockdown of TET1, TET2 or TET3 promoted neuronal differentiation of Neuro2a cells, and their overexpression inhibited VPA (valproic acid)-induced neuronal differentiation, suggesting all three TET proteins negatively regulate neuronal differentiation of Neuro2a cells. Interestingly, the inducing activity of TET protein is independent of its enzymatic activity. Our previous studies have demonstrated that srGAP3 can negatively regulate neuronal differentiation of Neuro2a cells. Furthermore, we revealed that TET1 could positively regulate srGAP3 expression independent of its catalytic activity, and srGAP3 is required for TET-mediated neuronal differentiation of Neuro2a cells. The results presented here may facilitate better understanding of the role of TET proteins in neuronal differentiation, and provide a possible therapy target for neuroblastoma.

**Electronic supplementary material:**

The online version of this article (doi:10.1007/s13238-016-0267-4) contains supplementary material, which is available to authorized users.

## INTRODUCTION

**Figure 1:**
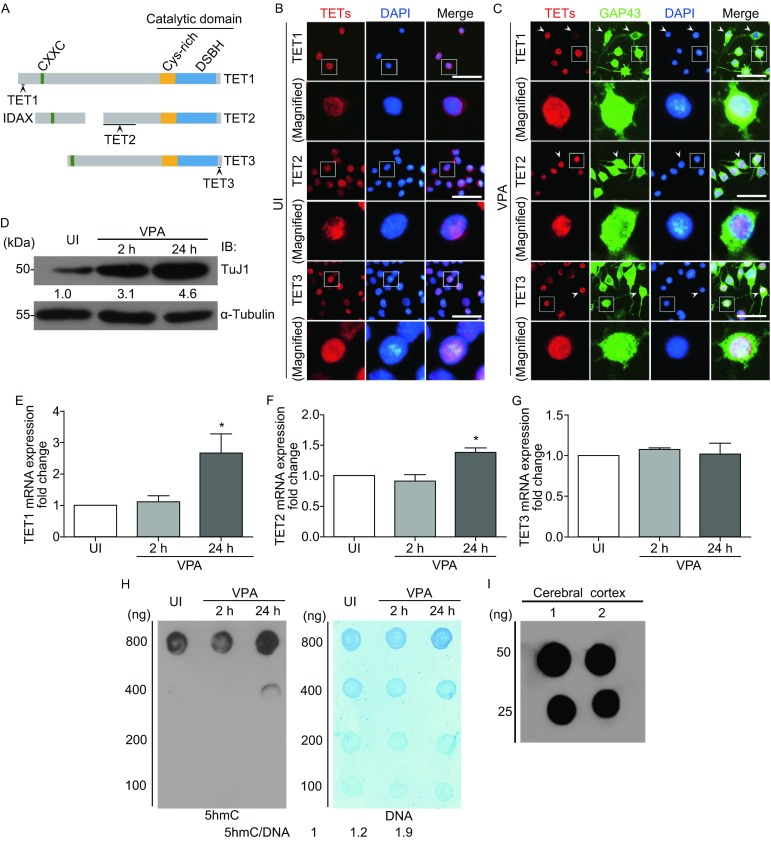
**Lower 5hmC level and higher expression of TET proteins in Neuro2a cells**. (A) Schematic presentation of the structure of TET proteins. The antigenic epitopes of the three TET antibodies on the corresponding TET proteins are underlined. (B) The expression and localization of endogenous TET proteins in Neuro2a cells cultured in high glucose DMEM containing 10% FBS (Uninduced, UI). The lower panels show magnifications of the single cell and process outlined by the white box in the upper panels respectively. Scale bar = 100 μm. (C) The expression and localization of endogenous TET proteins in opti-MEM containing 1 mmol/L VPA (VPA-induced, VPA) for 24 h. GAP43 was used as a neuronal differentiation marker. The lower panels show magnifications of the single cell and process outlined by the white box in the upper panels respectively. Scale bar = 100 μm. (D) Western blotting was used to detect the expression level of TuJ1 in uninduced Neuro2a cells (UI), and Neuro2a cells after VPA stimulation (VPA) for 2 h or 24 h. The relative expression level of TuJ1 was calculated by TuJ1/α-Tubulin. (E–G) Quantification of relative mRNA levels of endogenous TET proteins in uninduced Neuro2a cells (UI) and Neuro2a cells after VPA stimulation (VPA) for 2 h or 24 h. GAPDH was used as internal control. (H) Dot blotting was performed to analyze the 5hmC level in uninduced Neuro2a cells (UI), and Neuro2a cells after VPA stimulation (VPA) for 2 h or 24 h. DNA samples were serially two-fold diluted from 800 ng to 100 ng. 5hmC level was detected by using anti-5hmC antibody and the total DNA was stained by methylene blue. The relative level of 5hmC was calculated by 5hmC/DNA. (I) Dot blotting was used to analyze the 5hmC level in P15 mouse cerebral cortex tissues

DNA methylation at the 5-position of cytosine (5-methylcytosine, 5mC) is an important epigenetic mark involved in regulation of gene expression. Ten-eleven translocation proteins (TET1, TET2, and TET3) are a family of Fe (II) and α-ketoglutarate-dependent dioxygenases that are capable of oxidizing 5mC to 5-hydroxymethylcytosine (5hmC) and further to generate 5-formylcytosine (5fC) and 5-carboxylcytosine (5caC) (Pastor et al., 2013; Ito et al., 2011). TET family proteins share highly homologous protein structural features. All TET proteins contain a C-terminal double stranded β-helix catalytic domain, and both TET1 and TET3 have an N-terminal CXXC domain, while TET2 CXXC domain IDAX (also known as CXXC4) is separated from TET2 following chromosomal rearrangement during evolution (Fig. 1A).

The TET family members were identified to possess a catalytic enzymatic activity conversion 5mC to 5hmC in 2009 (Tahiliani et al., 2009). TET proteins had been proved to be critical for numerous biological processes such as embryonic development, stem cell differentiation, immune regulation, and cancer formation (Tan and Shi, 2012; Xu et al., 2012; Li et al., 2015; Zhang et al., 2015; Dawlaty et al., 2013; Cimmino et al., 2015; Neri et al., 2015; Scourzic et al., 2015). Importantly, 5hmC was first to identify highly enriched in Purkinje cells and the brain tissues, suggesting a potential biological function of 5hmC in these cells and tissues (Kriaucionis and Heintz, 2009). Indeed, accumulating evidence demonstrates that TET proteins play important roles in the regulation of neural function (Yao and Jin, 2014). For example, TET1 deficiency in mice results in impaired neurogenesis and memory (Zhang et al., 2013; Kaas et al., 2013); knockdown of TET2 and TET3 in the mouse cerebral cortex leads to a block in neuronal differentiation (Hahn et al., 2013); TET3 is essential for early eye development in *Xenopus* (Xu et al., 2012). The roles of TET proteins in transcriptional regulation have been extensively investigated (Pastor et al., 2013). In most cases, TET-mediated promoter hypomethylation facilitates gene expression (Ficz et al., 2011; Mariani et al., 2014; Wu et al., 2011) in a dioxygenase activity-dependent manner.

Besides the catalytic domains, the CXXC domains are also involved in TET-mediated gene expression regulation. The CXXC domains are important for TET proteins binding to specific genomic regions for their action (Xu et al., 2012; Tan and Shi, 2012; Jin et al., 2014), and they can cooperate with the catalytic domain to regulate the key gene expression (Xu et al., 2012; Ko et al., 2013). Interestingly, accumulating evidence suggests that the non-catalytic TET proteins also play important roles in regulating gene expression (Pastor et al., 2013), whereas the regulation mechanisms are far from being fully elucidated.

Neuro2a is a mouse neural crest-derived cell line that has been widely used as an experimental model for neuronal differentiation study. In our previous studies, we used this model to study the role of srGAP3 in neuronal differentiation, and we found srGAP3 negatively regulated valproic acid (VPA)-induced neuronal differentiation of Neuro2a cells (Chen et al., 2011; Ma et al., 2013). In this study, we investigated the role of TET proteins during neuronal differentiation using Neuro2a cells as a model. We found that all three TET proteins could negatively regulate neuronal differentiation of Neuro2a cells. Furthermore, TET1 can negatively modulate neuronal differentiation independent of its catalytic activity and through srGAP3.

## RESULTS

### The expression of TET proteins is not correlated with 5hmC level in Neuro2a cells

To investigate the roles of TET proteins in neuronal differentiation, we firstly detected TET1–3 expression in Neuro2a cells. Three polyclonal antibodies specific against TET1, TET2, and TET3 protein were applied in the study (Fig. 1A). Immunofluorescence staining was performed to visualize the subcellular localization of endogenous TET proteins (Fig. 1B and 1C). It could be clearly observed that all three TET proteins expressed at detectable levels and localized to the nuclei either in uninduced (UI) or VPA-induced (VPA) Neuro2a cells (Fig. 1B and 1C). TuJ1 was used as a neuronal differentiation marker to indicate the differentiation stages (Fig. 1D). qRT-PCR indicated that the expression levels of TET1 and TET2 but not TET3 were remarkably increased after VPA stimulation for 24 h (Fig. 1E–G).

However, it was reported that 5hmC level is low in Neuro2a cells (Kriaucionis and Heintz, 2009), and this conclusion was confirmed in this study. 5hmC level could be detected by spotting as much as 800 ng DNA in Neuro2a cells (Fig. 1H), compared to only 25 ng DNA in mouse cerebral cortex tissues (Fig. 1I). In addition, 5hmC level increased gradually during VPA-induced Neuro2a cells differentiation (Fig. 1H). Those results indicated Neuro2a cells maintained high level of TET proteins and lower level of 5hmC. The mismatch between TET proteins and 5hmC suggested the catalytic activities of TET proteins might be suppressed in Neuro2a cells.

### Knockdown of endogenous TET proteins promote neuronal differentiation of Neuro2a cells

**Figure 2:**
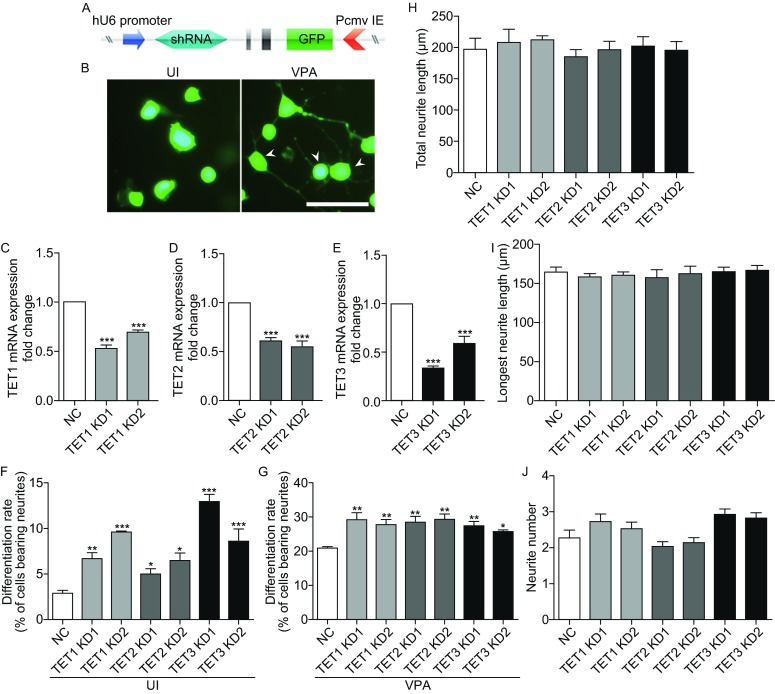
**Knockdown of TET proteins facilitate the neuronal differentiation of Neuro2a cells**. (A) Schematic presentation of shRNA expression vector. hU6 promoter, human U6 promoter; Pcmv IE, cytomegalovirus immediate-early promoter; GFP, green fluorescent protein. Neuro2a cells transfected with shRNA expression vectors were visualized by GFP. (B) Representative images of uninduced Neuro2a cells (UI) and Neuro2a cells after VPA stimulation (VPA) for 24 h. Arrowheads indicate the differentiated cells. Cells with processes longer than 1.5 cell bodies are considered as differentiated cells. Scale bar = 100 μm. (C–E) shRNA expression vectors of TET1–3 were transfected into Neuro2a cells, and qRT-PCR was used to analyze the effects of TET proteins knockdown. GAPDH was used as internal control. (F and G) Quantifications of the differentiation rates from Neuro2a cells of negative control (NC) and TET1, TET2, or TET3 knockdown in the absence (UI) or presence of VPA stimulation (VPA) for 24 h. Two shRNAs targeted different positions were designed respectively for each gene. (H–J) Assessment of neurite outgrowth of VPA-induced Neuro2a cells after TET1, TET2, or TET3 knockdown. Three endpoints were quantified: total neurite length (H), longest neurite length (I), and neurite number per cell (J). Values are mean ± SEM, *P < 0.05, **P < 0.01, ***P < 0.001

TET proteins play important roles in neuronal development; however, the regulatory mechanisms of TET family proteins remain largely unknown. Here we examined the effects of TET1, TET2, or TET3 depletion on Neuro2a cells by shRNA-based knockdown method. The plasmid pGPU6/GFP/Neo under the control of hU6 promoter and cytomegalovirus immediate-early promoter (Pcmv IE) was used to express shRNA and GFP, respectively (Fig. 2A). The Neuro2a cells transfected with either negative control or shRNA expressing vectors could be recognized by expression of GFP. Cells with neurite processes longer than 1.5 cell bodies were counted as differentiated cells (Fig. 2B). qRT-PCR analysis demonstrated the efficiency of knockdown (Fig. 2C–E). We then examined the effects of TET proteins knockdown on Neuro2a cells differentiation. As shown in Fig. 2F–G, TET proteins depletion promoted neuronal differentiation in Neuro2a cells. The differentiation rate of the two TET1 knockdown groups (TET1 KD1 and TET1 KD2) were 6.7% and 9.6%, respectively, in uninduced Neuro2a cells (UI) compared to the control group (NC) which was 2.9% (Fig. 2F), and were 29.2% and 27.8% in VPA-induced Neuro2a cells (VPA), respectively, compared to the control group, which was 20.9% (Fig. 2G). Additionally, similar effects on neuronal differentiation in Neuro2a cells could be observed after knockdown of TET2 (TET2 KD1 and TET2 KD2) or TET3 (TET3 KD1 and TET3 KD2) (Fig. 2F–G).

Furthermore, we tested whether knockdown of TET proteins could affect the neurite outgrowth of VPA-induced Neuro2a cells. The results showed that knockdown of TET1, TET2, or TET3 did not affect total neurite length (Fig. 2H), longest neurite length (Fig. 2I), and neurite number (Fig. 2G), suggesting that TET proteins could not alter neurite outgrowth in differentiated Neuro2a cells.

### Overexpression of TET proteins inhibit neuronal differentiation of VPA-induced Neuro2a cells

**Figure 3:**
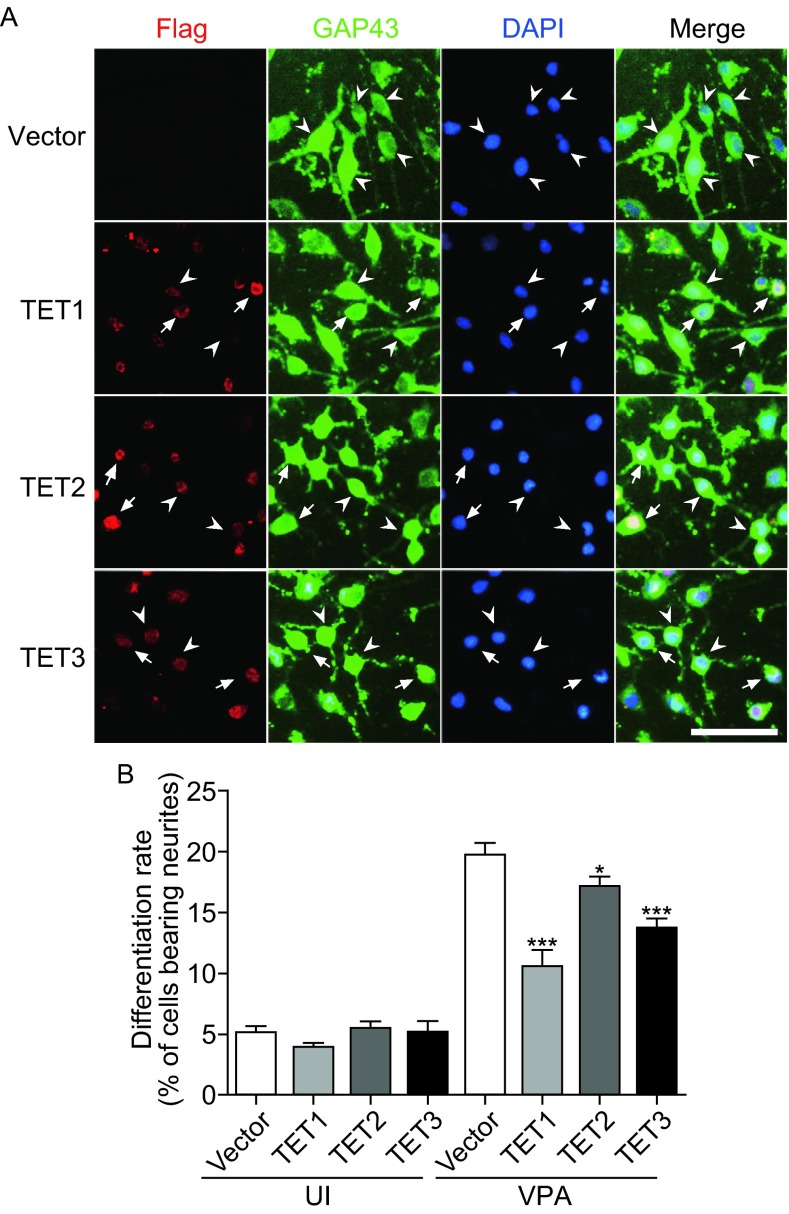
**Overexpression of TET proteins inhibits VPA-induced neuronal differentiation of Neuro2a cells**. (A) Neuro2a cells transfected with control vector (Vector) or vector harboring TET1, TET2, or TET3 gene were co-immunostained with Flag and GAP43 antibodies. Arrowheads indicate the differentiated cells and arrows indicate the undifferentiated cells. Scale bar = 100 μm. (B) Quantifications of the differentiation rates from Neuro2a cells in the absence (UI) or presence of VPA stimulation (VPA) for 24 h. Values are mean ± SEM, *P < 0.05, ***P < 0.001

We next tested whether overexpression of TET proteins could affect neuronal differentiation of Neuro2a cells. The plasmid harboring TET1, TET2, or TET3 gene was transfected into Neuro2a cells (Fig. 3A). TET1 overexpression in Neuro2a cells had an approximately 47.0% inhibitory effect on neuronal differentiation by VPA stimulation (Fig. 3B). The differentiation rate was reduced to 10.5% compared to control, which was 19.8% (Fig. 3B). Similarly, overexpression of TET2 or TET3 also leads to a remarkable reduction in differentiation rates (TET2 overexpression: 17.1%; TET3 overexpression: 13.7%) (Fig. 3B). The results demonstrated that TET family proteins are negative regulators in neuronal differentiation of Neuro2a cells.

### TET proteins enrich 5hmC in Neuro2a cells

**Figure 4:**
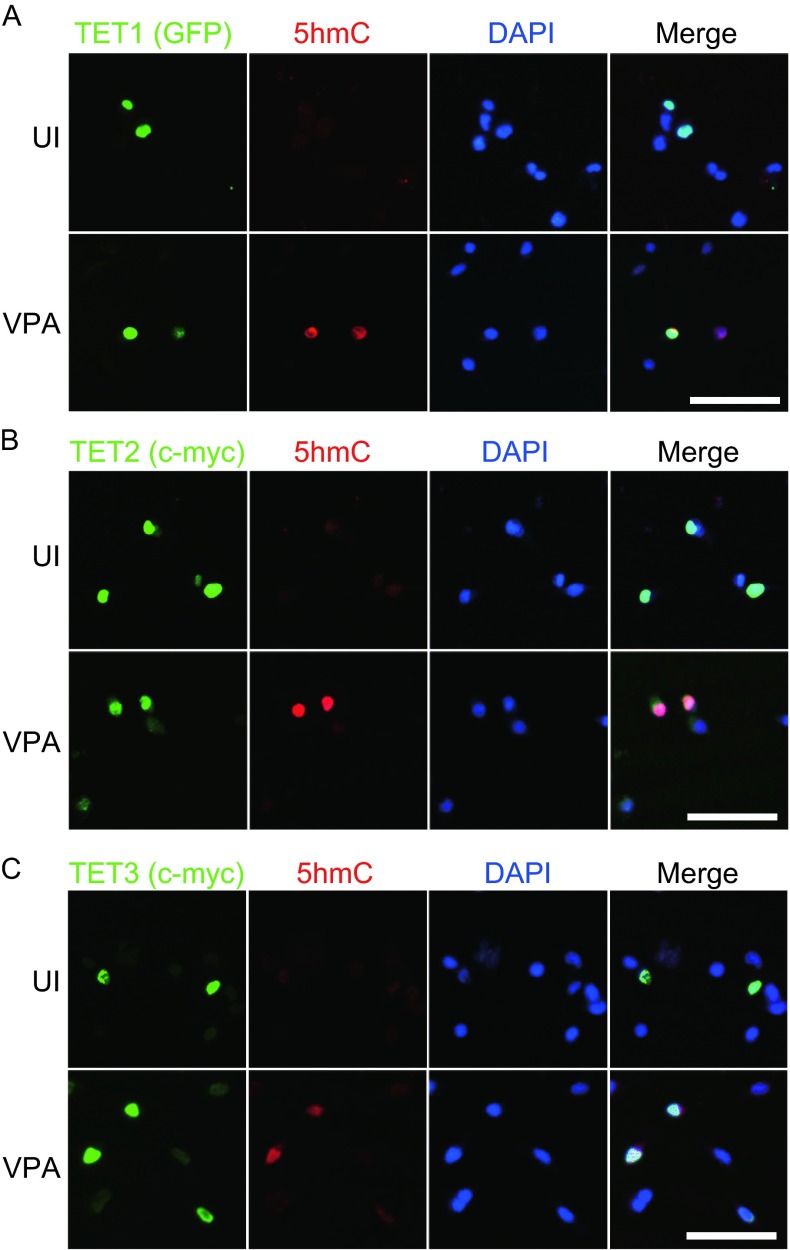
**Overexpression of TET proteins increased 5hmC levels in differentiated Neuro2a cells**. (A–C) Neuro2a cells were cultured in high glucose DMEM containing 10% FBS (UI) or in opti-MEM containing 1 mmol/L VPA (VPA). 5hmC and the expression of GFP tagged TET1 (A), c-myc tagged TET2 (B), or c-myc tagged TET3 (C) was detected by co-immunofluorescence staining

It is well known that TET proteins are able to convert 5mC to 5hmC, and enrich 5hmC in the cells. Given a mismatch between TET proteins and 5hmC levels (Fig. 1), we asked whether the overexpressed TET proteins kept their catalytic activities in the Neuro2a cells. As shown in Figure. 4, overexpression of TET1, TET2, or TET3 in Neuro2a cells can slightly increase 5hmC level. Importantly, the 5hmC levels were further increased after VPA stimulation for 24 h. The results suggested their catalytic activities were retained after TET protein overexpression, and the activities might be enhanced by VPA stimulation. However, whether this activity was involved in the regulation of neuronal differentiation of Neuro2a cells was unknown.

### Overexpression of non-catalytic TET1 inhibits Neuro2a cells differentiation

**Figure 5:**
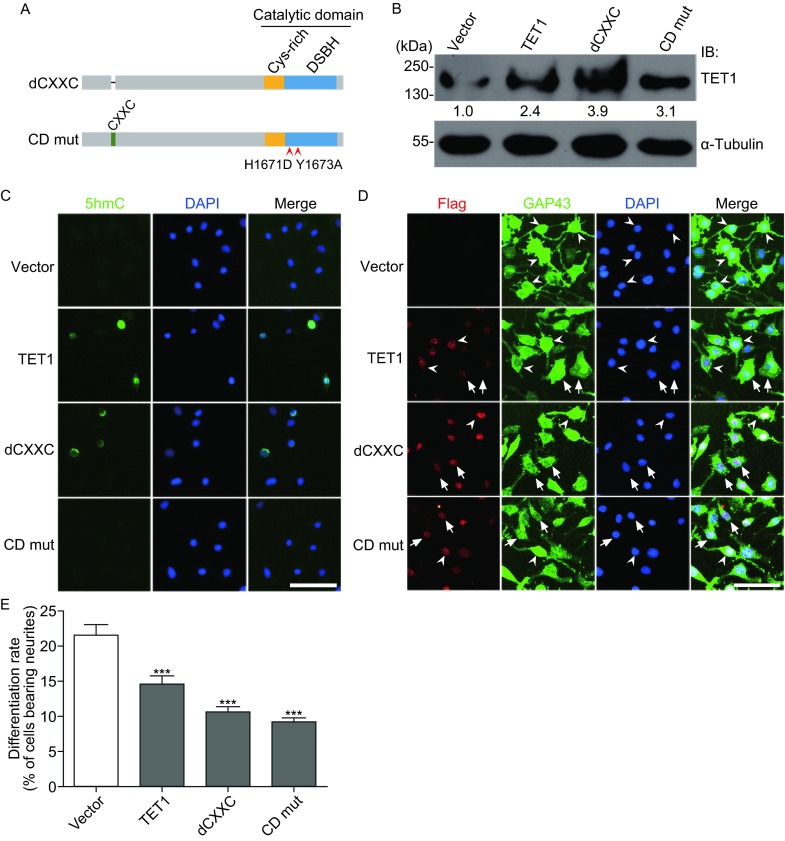
**Overexpression of TET1 mutants inhibits neuronal differentiation of Neuro2a cells**. (A) Schematic presentation of structure of TET1 mutants. (B) Neuro2a cells transfected with control vector (Vector) or vector harboring WT TET1 (TET1), TET1 dCXXC (dCXXC), and TET1 CD mut (CD mut) were lysed and immunoblotted with TET1 antibody. (C) Immunostaining of 5hmC after overexpression of WT TET1 (TET1), TET1 dCXXC (dCXXC), and TET1 CD mut (CD mut). (D) Neuro2a cells transfected with control vector (Vector) or vector harboring WT TET1 (TET1), TET1 dCXXC (dCXXC), and TET1 CD mut (CD mut) gene were co-immunostained with Flag and GAP43 antibodies. Arrowheads indicate the differentiated cells and arrows indicate undifferentiated cells. Scale bar = 100 μm. (E) Quantification of the differentiation rates from Neuro2a cells after overexpression of WT TET1 (TET1), TET1 dCXXC (dCXXC), and TET1 CD mut (CD mut). Values are mean ± SEM, ***P < 0.001

As TET1, TET2, and TET3 showed similar effect on Neuro2a differentiation, and all of them shared similar homologous regions, we chose TET1 as a representative for further study. While both dioxygenase and CpG binding activity are critical for TET1 function, it has been reported that TET1 could exert its effect through enzymatic activity-independent manner (Kaas et al., 2013; Wu et al., 2011; Williams et al., 2011). To explore whether the regulation of Neuro2a cells differentiation by TET1 is dependent on its enzymatic activity, we also tested the effects of inactive mutants of TET1 with CpG binding domain CXXC deletion mutant (dCXXC) and catalytic domain mutant (CD mut), respectively (Fig. 5A). The plasmids harboring wild type (WT) TET1, TET1 dCXXC (dCXXC), or TET1 CD mut (CD mut) genes, were transfected into Neuro2a cells. Western blotting confirmed the expression of those proteins (Fig. 5B), and immunocytochemistry confirmed that CD mut lost its catalytic activity, while the dCXXC retained it (Fig. 5C).

Next, we investigated the effects on neuronal differentiation in VPA-induced Neuro2a cells following transfection of these three plasmids. Interestingly, transfection of all three constructs including WT TET1 (TET1), dCXXC, and CD mut significantly inhibited Neuro2a cells differentiation (Fig. 5D and 5E), suggesting that TET1 regulated neuronal differentiation of Neuro2a cells in an enzymatic activity-independent manner.

### TET1 positively regulates srGAP3 expression independent of its catalytic domain

**Figure 6:**
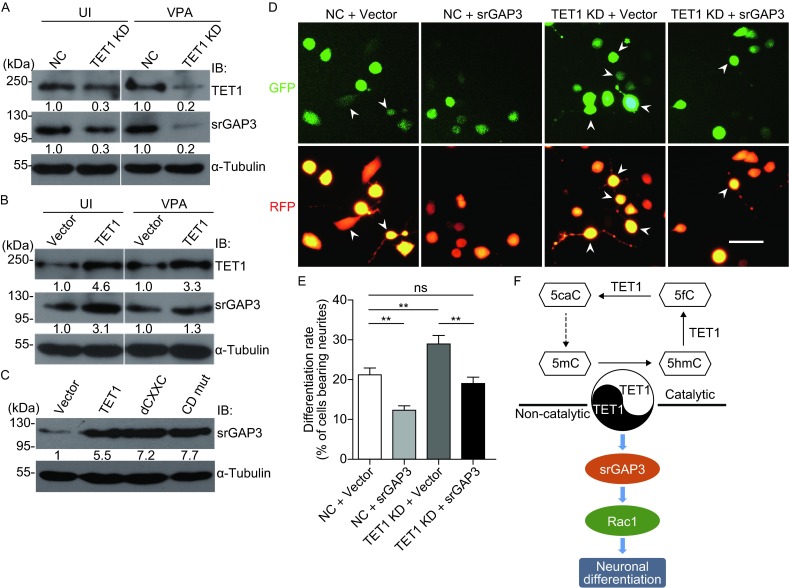
**TET1 inhibits neuronal differentiation of Neuro2a cells independent of catalytic activity through srGAP3**. (A) Western blotting was used to measure the srGAP3 level after endogenous TET1 knockdown in uninduced Neuro2a cells (UI), and Neuro2a cells after VPA stimulation (VPA) for 24 h. (B) Western blotting was used to measure the srGAP3 level after TET1 overexpression in uninduced Neuro2a cells (UI), and Neuro2a cells after VPA stimulation (VPA) for 24 h. (C) Western blotting was used to analyze srGAP3 level after overexpression of WT TET1 (TET1), TET1 dCXXC (dCXXC), and TET1 CD mut (CD mut). The relative expression level of srGAP3 was calculated by srGAP3/α-Tubulin. (D) SrGAP3 overexpression constructs or control vector and RFP were co-transfected into the normal or TET1-depleted Neuro2a cells, which were induced by VPA for 24 h. Arrowheads indicate the differentiated cells and arrows indicate undifferentiated cells. Scale bar = 100 μm. (E) Cell differentiation rate in (C) was analyzed. (F) A proposed working model for TET1 functions in regulating neuronal differentiation. Catalytic activity of TET1 might be suppressed in Neuro2a cells, and non-catalytic activity of TET1 inhibited neuronal differentiation through srGAP3 and Rac1 pathway. Values are mean ± SEM, **P < 0.01

Since TET1 has been demonstrated to regulate a series of gene expression, we asked whether TET1 could modulate srGAP3 expression. We examined srGAP3 expression after TET1 knockdown and overexpression by Western blotting. TET1 KD1, one of the two shRNA expressing vectors, which was more effective than the other, was selected for subsequent research. Indeed, the results showed that knockdown of endogenous TET1 by transfection of shRNA against TET1 decreased protein levels of srGAP3 either in uninduced (UI) or VPA-induced (VPA) Neuro2a cells (Fig. 6A), while overexpression of TET1 increased srGAP3 expression (Fig. 6B). In our previous studies, srGAP3 was illustrated to negatively regulate neuronal differentiation of VPA-induced Neuro2a cells in a Rac1-dependent manner (Chen et al., 2011; Ma et al., 2013). Thus, we speculated that TET1 might negatively regulate neuronal differentiation of Neuro2a cells via modulating the expression of srGAP3. Considering that wild type (WT) TET1 can positively regulate the srGAP3 expression, we wonder whether the TET1 mutants can also modulate the srGAP3 expression. We examined the srGAP3 expression level following overexpression of WT TET1 (TET1), dCXXC, and CD mut in Neuro2a cells. As shown in Figure 6C, overexpression of WT TET1 (TET1), dCXXC, and CD mut can upregulate srGAP3 expression.

To confirm if srGAP3 can reverse the neuronal differentiation inducing activity of TET1-depleted neuro2a cells, we then quantified the differentiation rate of Neuro2a cells co-transfected with the knockdown construct for TET1 and srGAP3 overexpression vectors. As expected, overexpression of srGAP3 efficiently attenuated VPA-induced neuronal differentiation of TET1-depleted Neuro2a cells (Fig. 6D and 6E), indicating that TET1 negative regulate neuronal differentiation through srGAP3 expression, independent of its enzymatic activity (Fig. 6F).

## DISCUSSION

Accumulating evidence indicates that TET proteins regulate neuronal activity-related gene expression, neuronal survival, neural development, and memory formation (Zhang et al., 2013; Kaas et al., 2013; Rudenko et al., 2013). In this study, we employed a Neuro2a cell model to explore the effect of TET family proteins on neural differentiation *in vitro*. We demonstrated TET proteins could negatively regulate neuronal differentiation of Neuro2a cells, and this regulation is independent of enzymatic activity of TET1. Furthermore, non-catalytic TET1 could negatively regulate neuronal differentiation through upregulating srGAP3.

Both the catalytic domain and CXXC domain are important for TET protein enzymatic activity. Mutations of the TET catalytic domain and CXXC domain can reduce their enzymatic-activity-dependent function (Hsu et al., 2012). Besides functioning in an enzymatic dependent manner, TET proteins can also regulate gene transcription independent of their catalytic activities. For examples, TET1 is found to repress gene transcription through binding to Polycomb group target genes, which is independent of its catalytic activity (Williams et al., 2011; Wu et al., 2011), and it can also upregulate several neuronal memory-associated genes independent of its enzymatic activity (Kaas et al., 2013). Similarly, TET2 was identified to recruit histone deacetylases 2 (Hdac2) to repress IL-6 transcription independent of DNA methylation and hydroxymethylation (Zhang et al., 2015). Additionally, catalytically inactive TET3 mutant can partially rescue the effect on *Xenopus* eye and neural development after TET3 depletion, suggesting an enzymatic activity-independent mechanism in gene regulation (Xu et al., 2012). These findings point out a critical role of TET-mediated gene regulation independent of enzymatic activity. In this study, we uncovered that the differentiation rate was significantly decreased after overexpression of both the TET1 dCXXC and TET1 CD mutant, showing that the neuronal differentiation of Neuro2a cells was negatively regulated by TET1 in a dioxygenase activity-independent manner. Furthermore, overexpression of TET1 mutants resulted in a stronger inhibitory effect on neuronal differentiation even than that of overexpression of WT TET1 (TET1) (Fig. 5E). That might be because WT TET1 may exert an opposite effect on neuronal differentiation in an enzymatic activity-dependent manner.

TET1 can regulate the srGAP3 expression, and this regulation is independent of the catalytic activity of TET1. As srGAP3 can negatively regulate neuronal differentiation of Neuro2a cells, we speculate TET1 may negatively regulate Neuro2a cells differentiation via modulating the expression of srGAP3. Since many proteins have been found to interact with TET proteins to regulate gene expression (Yao and Jin 2014; Qiao et al., 2015; Wang et al., 2015; Perera et al., 2015), the regulation of srGAP3 expression may be due to the interaction between TET1 with some unknown proteins. However, the precise mechanism of neuronal differentiation regulated by TET1 should be further investigated.

In addition, the mRNA levels of TET1 and TET2 were increased after VPA stimulation for 24 h (Fig. 1E and 1F). It has been reported that VPA inhibit histone deacetylases (HDACs) to relieve HDAC-mediated transcriptional repression (Göttlicher et al., 2001). Thus, the increased mRNA of TET1 and TET2 may be due to the increase of histone acetylation level by VPA treatment. Furthermore, 5hmC levels were also increased in VPA-induced Neuro2a cells. These results were consistent with previous observations that levels of 5hmC increased during neuronal differentiation (Hahn et al., 2013). It also suggested the inhibition of catalytic activity of TET proteins might be relieved during neuronal differentiation of Neuro2a cells.

Neuroblastoma is the most common extracranial solid tumor of early childhood. Induction of terminal differentiation is a therapeutic approach for neuroblastoma treatment (Brodeur and Bagatell, 2014). In this study, we found TET proteins could negatively regulate neuronal differentiation of mouse neuroblastoma Neuro2a cells. It suggested inhibition of TET protein expression might be a promising approach for neuroblastoma therapy. Furthermore, in a previous report, it was suggested that hypoxia leads to transcriptional activation of TET1, which facilitated hypoxic gene induction in neuroblastoma (Mariani et al., 2014), showing the important role of TET1 in neuroblastoma development. The results further indicated TET1 might serve as a promising therapeutic target in neuroblastoma.

In summary, we found TET1 could negatively regulate neuronal differentiation of Neuro2a cells independent of its enzymatic activity through srGAP3. This study might provide a better understanding for the role of TET proteins in neuronal differentiation and offer a new potential therapy for neuroblastoma.

## MATERIALS AND METHODS

### Antibodies

Three homemade TET1 (from 146 to 159 amino acid residues), TET3 (from 1598 to 1610 amino acid residues), and srGAP3 (from 1088 to 1099 amino acid residues) antigen affinity antibodies were used in our previous studies (Zhao et al., 2014; Mi et al., 2015; Fu et al., 2014). These antibodies were purified by antigen affinity column. Rabbit anti-TET2 antibody was purchased from Proteintech Group (Chicago, USA). All of these antibodies could specifically recognize the corresponding proteins in immunofluorescence and Western blotting (Zhao et al., 2014; Mi et al., 2015; Fu et al., 2014). Rabbit anti-5hmC antibody (#39769) was purchased from Active Motif (Carlsbad, USA).

### Quantitative RT-PCR

To obtain total RNA, Neuro2a cells were extracted using Trizol Reagent (Invitrogen, Carlsbad USA). The RNA was subsequently treated with RNase-free DNase I (Roche, Mannheim, Germany). The isolated total RNA was reverse-transcribed for two-step quantitative RT-PCR (qRT-PCR) using the PrimeScript RT reagent kit (TAKARA, Kyoto, Japan) according to the manufacturer’s instructions. Quantitative RT-PCR was carried out using a Peltier Thermal Cycler (BioRad, Hercules, USA) plus Real time PCR Master Mix (SYBR Green, Toyobo, Japan). Gene-specific primer pairs for PCR are listed in Table S1. GAPDH was served as an internal control.

### Plasmids

According to the target sequences of TET1, TET2, and TET3, oligonucleotides encoding shRNA were designed, synthesized, and inserted into pGPU6/GFP/Neo vector (Genepharma, Shanghai, China) to generate recombinant plasmids expressing shRNAs against TET1, TET2, and TET3, respectively. The target sequences for shRNAs are listed in Table S2. The recombinant plasmids expressing full-length TET1, inactive mutant of catalytic mutant (CD mut), and CXXC-deleted mutant (dCXXC) of TET1 were kindly provided by Dr. Li-Jung Juan (Hsu et al., 2012). The recombinant plasmids expressing full-length TET2 and full-length TET3 were obtained from Dr. Ross L. Levine (Abdel-Wahab et al., 2009) and Dr. Toshinobu Nakamura (Nakamura et al., 2012).

### Cell culture, transfection, and differentiation assay

Neuro2a cells were cultured in DMEM containing 10% fetal bovine serum (FBS), 1% non-essential amino acid, and 1% penicillin and streptomycin. Neuro2a cells were transfected using X-treme GENE HP DNA Transfection Reagent (Roche, Mannheim, Germany) according to the manufacturer’s instructions. To induce neuronal differentiation, Neuro2a cells (about 20% confluences) were transferred to serum-free opti-MEM (Gibco, Grand Island, New York, USA) containing 1 mmol/L VPA for neurite extension.

### Immunocytochemistry

Cells on Poly-L-Lysine-coated glass coverslips were fixed with 4% paraformaldehyde for 15 min at room temperature and then permeabilized by treatment with ice-cold methanol for 10 min. For staining 5hmC, DNA was denatured with 2 mol/L HCl for 30 min, and then neutralized with 0.1 mol/L Tris-HCl (pH 8.5) twice (10 min a time). Subsequently, the cells were blocked by 15% normal donkey serum for 30 min, and then were incubated at 4°C overnight with primary antibody. The following antibodies dilutions were used: all TET antibodies were used at 1:200, anti-5hmC (1:5000), anti-GAP43 (1:500), and anti-Flag (PTGlab; 1:200). Then, the cells were rinsed and incubated for 2 h with fluorescence-labeled secondary antibodies. After washing, the coverslips were mounted with PBS containing 50% glycerol and 5 μg/mL DAPI for nuclei staining.

### Dot blotting

The genomic DNA of Neuro2a cells were isolated using the EZNA Tissue DNA kit (OMEGA, Norcross, USA) and the DNA concentration was measured by NanoDrop. Dot-blot assays have been described previously (Ito et al., 2011). Briefly, genomic DNA was spotted on nylon membrane, and then baked at 80°C for 2 h. After blocked with 5% skimmed milk for 1 h, nylon membrane was incubated with the anti-5hmC antibody (1:5000) overnight at 4°C, and then incubated with POD-labeled secondary antibodies (1:12500; Roche, Mannheim, Germany). The signals were detected by BM Chemiluminescence Western Blotting kit (Roche, Mannheim, Germany). The densitometry quantification of dot-blot was analyzed by ImageProPlus 6.0.

### Western blotting

The procedure had been previously described (Chen et al., 2011). In brief, the cells were lysed and the protein concentrations of the lysates were determined. Then, the lysates were separated by 8% SDS-PAGE and transferred to polyvinylidene fluoride (PVDF) membranes. The membranes were blocked, followed by incubation with primary antibodies and then POD-labeled secondary antibodies (1:12500; Roche, Mannheim, Germany). The primary antibodies were used as follows: TuJ1 (1:1000), TET1 (1:500), srGAP3 (1:1000), and α-Tubulin (Santa Cruz; 1:5000). The signals were detected by BM Chemiluminescence Western Blotting kit (Roche, Mannheim, Germany).

### Statistical analysis

For assessing differentiation, cells bearing neurite processes 1.5 times longer than cell bodies were considered to be differentiated. Each group was analyzed by counting at least 300 cells. Assessment of neurite outgrowth was evaluated by counting about 30 to 50 cells per condition. Neurite number and length were quantified using Image Pro-Plus software. In each analysis, the data represent mean ± SEM of at least three experiments. For comparison, statistical significance was tested by one-way ANOVA.

## Electronic supplementary material

Below is the link to the electronic supplementary material.

Supplementary material 1 (PDF 8 kb)
