# Supplementary material for: Non-catalytic roles for TET1 protein negatively regulating neuronal differentiation through srGAP3 in neuroblastoma cells
Source: Protein Cell. 2016 Apr 25;7(5):351–61. doi: 10.1007/s13238-016-0267-4 (PMC4853314; doi:10.1007/s13238-016-0267-4)
Supplement: Supplementary file 1 — Supplementary material 1 (PDF 8 kb) [file 13238_2016_267_MOESM1_ESM.pdf]

Supplementary Table 1. Primer sequences for target genes

Gene name	Target position	Accession No.	Primer sequence	Expect size
TET1	5642 bp – 5788 bp	NM-001253857	5’-ATGAGCGGCACCCTGAAGCG-3’<br>5’-GCACCGAGCCGTGAATGGGT-3’	147bp
TET2	4319 bp – 4422 bp	NM_001040400	5’- AGAGAAGACAATCGAGAAGTCGG-3’<br>5’-CCTTCCGTACTCCCAAACTCAT-3’	104bp
TET3	4367 bp – 4471 bp	NM_183138.2	5’-CACGGCTTCGAGGCAAGCCA-3’<br>5’-CCCCGGTTCCCATCCCCCAT-3’	105bp

Supplementary Table 2. ShRNA sequences

Name	Target sequence
Negative control	5'-TTCTCCGAACGTGTCACGT-3'
TET1 KD1[a]	5'-GCTCATGGAGACTAGGTATGG-3'
TET1 KD2[a]	5'-GCAGATGGCCGTGACACAAATT-3'
TET2 KD1[b]	5'-GGGTAAGCCAAGAAAGAAA-3'
TET2 KD2	5'-CCAGGGATGTCCTATTGCTAAA-3'
TET3 KD1	5'-ACCGCATCTCGCTGGTCTTCTA-3'
TET3 KD2[b]	5'-GCTCCAACGAGAAGCTATTTG-3'

REFERENCES:

a. Li T, Yang D, Li J, Tang Y, Yang J, Le W (2015) Critical role of Tet3 in neural progenitor cell maintenance and terminal differentiation. Mol Neurobiol 51 (1):142-154

Williams K, Christensen J, Pedersen MT, Johansen JV, Cloos PA, Rappsilber J, Helin K (2011) TET1 and hydroxymethylcytosine in transcription and DNA methylation fidelity. Nature 473 (7347):343-348

b. Pronier E, Almire C, Mokrani H, Vasanthakumar A, Simon A, Mor BdCRM, Massé A, Le Couédic J-P, Pendino F, Carbonne B (2011) Inhibition of TET2-mediated conversion of 5-methylcytosine to 5-hydroxymethylcytosine disturbs erythroid and granulomonocytic differentiation of human hematopoietic progenitors. Blood 118 (9):2551-2555

.
